# Essential oil from *Xylopia frutescens* Aubl. reduces cytosolic calcium levels on guinea pig ileum: mechanism underlying its spasmolytic potential

**DOI:** 10.1186/s12906-015-0849-3

**Published:** 2015-09-16

**Authors:** Iara Leão Luna de Souza, Ana Carolina de Carvalho Correia, Layanne Cabral da Cunha Araujo, Luiz Henrique César Vasconcelos, Maria da Conceição Correia Silva, Vicente Carlos de Oliveira Costa, Josean Fechine Tavares, Edgar Julian Paredes-Gamero, Fabiana de Andrade Cavalcante, Bagnólia Araújo da Silva

**Affiliations:** Centro de Ciências da Saúde/Universidade Federal da Paraíba, João Pessoa, Paraíba Brazil; Instituto de Ciências Biológicas e da Saúde/Universidade Federal de Alagoas, Maceió, Alagoas Brazil; Centro de Ciências Exatas e da Natureza/Universidade Federal da Paraíba, João Pessoa, Paraíba Brazil; Instituto de Pesquisa em Fármacos e Medicamentos/Universidade Federal da Paraíba, João Pessoa, Paraíba Brazil; Instituto Nacional de Farmacologia/Universidade Federal de São Paulo, São Paulo, São Paulo Brazil; Universidade Federal da Paraíba, Centro de Ciências da Saúde/Pós-Graduação em Produtos Naturais e Sintéticos Bioativos/Laboratório de Farmacologia Funcional Prof. George Thomas, Cidade, Universitária, P.O. Box 5009, 58051-970 João Pessoa, Paraíba Brazil

**Keywords:** *Xylopia frutescens*, Essential oil, Spasmolytic action, Guinea pig ileum, Calcium

## Abstract

**Background:**

*Xylopia frutescens* Aubl. (embira, semente-de-embira or embira-vermelha), is used in folk medicine as antidiarrheal. The essential oil from its leaves (XF-EO) has been found to cause smooth muscle relaxation. Thus, the aim of this study was to investigate the spasmolytic action by which XF-EO acts on guinea pig ileum.

**Methods:**

The components of the XF-EO were identified by gas chromatography-mass spectrometry. Segments of guinea pig ileum were suspended in organ bath containing modified Krebs solution at 37 °C, bubbled with carbogen mixture under a resting tension of 1 g. Isotonic contractions were registered using kymographs and isometric contractions using force transducer coupled to an amplifier and computer. Fluorescence measurements were obtained with a microplate reader using Fluo-4.

**Results:**

Forty-three constituents were identified in XF-EO, mostly mono- and sesquiterpenes. XF-EO has been found to cause relaxation on guinea pig ileum. The essential oil inhibited in a concentration-dependent manner both CCh- and histamine-induced phasic contractions, being more potent on histamine-induced contractions as well as antagonized histamine-induced cumulative contractions in a non-competitive antagonism profile. XF-EO relaxed in a concentration-dependent manner the ileum pre-contracted with KCl and histamine. Since the potency was smaller in organ pre-contracted with KCl, it was hypothesized that XF-OE would be acting as a K^+^ channel positive modulator. In the presence of CsCl (non-selective K^+^ channel blocker), the relaxant potency of XF-OE was not altered, indicating a non-participation of these channels. Moreover, XF-EO inhibited CaCl_2_-induced cumulative contractions in a depolarizing medium nominally without Ca^2+^ and relaxed the ileum pre-contracted with S-(-)-Bay K8644 in a concentration-dependent manner, thus, was confirmed the inhibition of Ca^2+^ influx through Ca_v_1 by XF-EO. In cellular experiments, the viability of longitudinal layer myocytes from guinea pig ileum was not altered in the presence of XF-OE and the Fluo-4-associated fluorescence intensity in these intestinal myocytes stimulated by histamine was reduced by the essential oil, indicating a [Ca^2+^]_c_ reduction.

**Conclusion:**

Spasmolytic action mechanism of XF-EO on guinea pig ileum can involve histaminergic receptor antagonism and Ca^2+^ influx blockade, which results in [Ca^2+^]_c_ reduction leading to smooth muscle relaxation.

## Background

Diarrhea is characterized by change in bowel movements with symptoms of increment in water content, volume and frequency of stool [1]. It is the second leading cause of death in children under five years old, and it is responsible for around 760.000 children deaths every year [2]. For this reason, international organizations have encouraged studies pertaining to treatment and prevention of diarrheal diseases using traditional medicinal practices [3].

*Xylopia frutecens* Aubl. is a tree commonly known in Brazil as “embira”, “semente-de-embira” and “embira-vermelha” [4] and belongs to the Annonaceae family. In folk medicine, its seeds are used to rheumatism and inflammation treatment, to improve digestion and as antidiarrheal [5–7].

Chemical studies showed that kaurenoic acid, a kaurene diterpene abundant in the seeds of this species presents anti-inflammatory [7], analgesic [8], diuretic and vasorelaxant activities [9]. In addition, pharmacological investigations have demonstrated antiviral [10], antifungal [11], tripanossomicide [12], antitumor [13] and spasmolytic activity [14] for *X. frutescens*. Despite the common use of this plant in popular medicine, there is a lack of information to support its effect on intestinal smooth muscle. Hence, the present study aimed to evaluate the spasmolytic activity and possible mechanism of action of the essential oil from *X. frutescens* (XF-EO) on guinea pig ileum, to ascertain ethnopharmacological claims.

## Methods

### Plant material and essential oil preparation

The plant material of *Xylopia frutescens* Aubl. was collected in April 2010 in João Pessoa municipality, State of Paraíba, Brazil. The material was identified by Maria de Fátima Agra (PhD) of the Centro de Biotecnologia (CBiotec) of Universidade Federal da Paraíba (UFPB), and the voucher specimen (Agra 7249) is deposited at the Prof. Lauro Pires Xavier (JPB) Herbarium/UFPB.

Fresh leaves from *Xylopia frutescens* were ground and submitted to hydrodistillation using a Clevenger-type apparatus (1500 g, 6 h, 40 °C). The oil was dried over anhydrous sodium sulfate and its percentage content was calculated on basis of the plant dry weight. The essential oil was used for the spasmolytic activity studies.

### Identification of essential oil components

The analysis of the essential oil was carried out on a SHIMADZU gas chromatography–mass spectrometry (GC-MS) instrument under the following conditions: DB-5 ms (30 m x 0.25 mm d.i., 0.25 μM film thickness), fused-silica capillary column, programmed temperature of 60-240 °C (3 °C/min), injector temperature at 220 °C, helium carrier gas adjusted to a linear velocity of 32 cm/s (measured at 100 °C), splitless injection (2 μL 1:1000 hexane solution), split flow adjusted to yield a 20:1 ratio, septum sweep constant at 10 mL/min, EIMS electron energy of 70 eV, ion source and connections at 200 °C. The quantitative data for the volatile constituents were obtained by peak-area normalization using a FOCUS GC/ flame ionization detector (FID), operated under GC-MS similar conditions except for the carrier gas, which was nitrogen. The retention index was calculated for all the volatile constituents using an n-alkane (C8-C20, Sigma-Aldrich, Brazil) homologous series.

Individual components were identified by comparison of both mass spectrum and GC retention data with previously analyzed authentic compounds stored in our private library, as well as with the aid of commercial libraries containing mass spectra, and retention indices of volatile compounds commonly found in essential oils [15, 16].

### Chemicals

The following reference chemicals were obtained from the sources specified: histamine dihydrochloride, carbachol (CCh) (Merck, EUA), Cremophor EL®, S-(-)-Bay K8644, cesium chloride (CsCl), EDTA, HEPES, methylthiotetrazole (MTT), dimethyl sulfoxide (DMSO), penicillin-streptomycin (Sigma-Aldrich, Brazil), ethanol PA (Reagen, Brazil), dulbecco’s modified eagle medium (DMEM), bovine fetal serum, L-glutamine and trypsin/EDTA solution (1:250) (Cultilab, Brazil) and Flou-4 NW dye mix (Invitrogen, EUA).

The following chemicals were used to make the physiological salt solutions: glucose, magnesium sulphate, calcium chloride (Vetec, Brazil), sodium bicarbonate (Fmaia, Brazil), sodium chloride, potassium chloride (Química Moderna, Brazil), monosodium phosphate-1-hydrate, sodium hydroxide and hydrochloric acid (Nuclear, Brazil)

All chemicals used were of the highest purity grade available. Stock solutions of all the chemicals were made in distilled water and the dilutions were made fresh on the day of the experiment.

Carbogen mixture (95 % O_2_ and 5 % CO_2_) was obtained from White Martins (Brazil).

### Animals and organ manipulation

Adult guinea pigs (*Cavia porcellus*) of both sexes weighing 300–500 g, obtained from Bioterium Prof. Thomas George of UFPB, were used in this study. Animals had full access to food (Labina®) and water, were kept in rooms at 21 ± 1 °C and submitted to a 12 h light-dark cycle (lights on 06 to 18 h). Eighteen hours before experiments, food was withheld and free access to water was maintained. All experimental procedures were performed in accordance with guidelines for the ethical use of animals in applied etiology studies [17] and approved by UFPB Ethic Committee on Animal Use (protocol/CEUA no. 0611/13).

Animals were euthanized by cervical dislocation. An ileum segment (approximately 15 cm) was removed, cleaned of adhering fat and connective tissue, and suspended in organ baths containing modified Krebs solution [18] at 37 °C and bubbled with carbogen mixture. Solution pH was adjusted to 7.4 with HCl or NaOH 1 N, as necessary. The resting tension was fixed at 1 g and the preparation was allowed to stabilization for 30 min.

To register isotonic contractions, organs were suspended by cotton yarn in organ baths (5 mL) and recorded on smoked drum through levers coupled to kymographs (DTF, Brazil). To register isometric contractions, organs were suspended in steel rods in organ baths (6 mL), connected to a force transducer (TIM 05), attached to an amplifier (AECAD04F) and connected to an A/D converter into a PC running AQCAD® software (São Paulo, Brazil).

### Cell culture

The guinea pig ileum was collected and the longitudinal smooth muscle layer was carefully stripped off. The pieces removed were placed in warmed physiological solution and the organ was successively washed with solution without Ca^2+^ and enriched with penicillin. Afterwards, tissue samples were transferred to culture bottles and was added DMEM culture medium supplemented with glutamine and 10 % bovine fetal serum, then, the bottles were stored in CO_2_ incubator [19]. After 24 h, the culture medium was added to the bottles and 48 h later the bottles were washed with PBS and the culture medium was replaced until the bottles become confluent. In this case, the medium was removed, added trypsin/EDTA (1:2500) for 2 min in incubator and the cells were resuspended in culture medium and centrifuged (500 × g) for 5 min. The supernatant was discarded and the pellets formed were used in the experiments.

### Spasmolytic analysis

#### Effect of XF-EO on carbachol- and histamine-induced phasic contractions

Two similar concentration-response curves were obtained with CCh (10^−6^ M) or histamine (10^-6^ M). Then, XF-EO effects were determined by pre-incubating the ileum strips for 15 min with a single concentration of the essential oil in independent experiments before adding CCh or histamine. The inhibitory effect exerted by the essential oil was evaluated based on IC_50_ analysis, which is defined as the molar concentration values of an antagonist which produces 50 % of its maximum inhibitory response, and maximum effect (E_max_), assessed through concentration-response curves in both absence (control) and presence of XF-EO [20].

#### Effect of XF-EO on histamine-induced cumulative contractions

Two similar cumulative concentration-response curves for histamine (10^-9^-10^-4^ M) were induced (control) and the tissue was exposed to different concentrations of XF-EO for 15 min, followed by a new histamine cumulative curve. The maximal contraction obtained in the absence of XF-EO was considered to be 100 % (control) and all contractions were assessed referring to it. The inhibitory effect exerted by the essential oil was evaluated based on analysis of EC_50_ values, which is defined as the molar concentration values of an agonist which produces 50 % of its maximum effective response, and E_max_, assessed through concentration-response curves in both absence (control) and presence of XF-EO [21].

#### Effect of XF-EO on KCl-induced tonic contraction or histamine-induced tonic contractions in both absence and presence of non-selective potassium channel blocker

A contraction was induced with KCl (40 mM) or histamine (10^−6^ M). During the tonic phase, XF-EO was cumulatively added as an attempt to obtain concentration-relaxation curves in different preparations. Then, the histamine-induced tonic contraction was washed out and CsCl (5 mM), a non-selective K^+^ channel blocker [22, 23], was added for 20 min. Then, another tonic contraction was elicited in the presence of the blocker and the essential oil was cumulatively added. The relaxant potency of XF-EO was evaluated by comparing EC_50_ values in both absence (control) and presence of CsCl.

#### Effect of XF-EO on Ca^2+^ influx

After stabilization period, the modified Krebs solution was replaced by a depolarizing modified Krebs solution nominally without Ca^2+^ (KCl, 4 mM was increased to 70 mM with equimolar exchange for NaCl). After 45 min, two concentration-response curves to CaCl_2_ (10^−5^ to 10^−1^ M) were constructed (control) and the tissue was exposed to different concentrations of XF-EO for 15 min, followed by a new concentration-response curve to CaCl_2_. The maximal contraction obtained in the absence of XF-OE was considered to be 100 % (control) and all contractions were assessed referring to it. The inhibitory effect exerted by the essential oil was evaluated based on analysis of EC_50_ and E_max_ values, assessed through concentration-response curves in both absence (control) and presence of XF-EO [24].

In other experiments, the ileum was partially depolarized with KCl (15 mM) for 10 min [25], then, was induced a contraction with S-(-)-Bay K8644 (3 x 10^-7^ M), a selective voltage-dependent calcium channel (Ca_v_) agonist to L-type or Ca_V_1 [26]. During the tonic contraction, the essential oil was added cumulatively in different preparations, in order to obtain a relaxation curve. The relaxation was expressed as the reversal percentage of initial contraction elicited by the S-(-)-Bay K8644 and evaluated by comparing EC_50_ values in both absence (control) and presence of the Ca_V_1 agonist.

#### Effect of XF-EO on myocytes from ileum longitudinal layer

##### Interference of XF-EO on ileum myocytes viability

Cell viability was determined using the MTT assay [27]. Briefly, ileum myocytes were seeded at the density of 70–80 % confluence per well in 96-well plates for 24 h incubation and treated with XF-EO. After treatment, 10 μL of MTT (5 mg/mL) was added and the myocytes were incubated for 6 h at 37 °C in CO_2_ incubator. The supernatant was discarded and the water-insoluble dark blue formazan crystals formed in viable cells were solubilized in DMSO. The spectrophotometric absorbance was measured at 540 nm using a microplate reader (FlexStation 3) and the Soft Max Pro software (Molecular Devices, USA). Triplicate experiments were performed and the absorbance from the wells of cells cultured with DMSO was taken as 100 % (control) of viability value.

##### Effect of XF-EO on cytosolic calcium concentration of ileum myocytes

The pellets were obtained as described earlier, cultured in 96-well plates (40.000 cells per well) and stabilized in incubator for 24 h for cell adhesion. After this process, the culture medium of each well was discarded and 50 μL of Fluo-4 (Molecular Probes/Invitrogen, USA) was added and let rest for 40 min at 37 °C in CO_2_ incubator [28]. After the fluorophore incorporation, the Fluo-4-associated fluorescence was quantified in a microplate reader (FlexStation 3) using the Soft Max Pro software (Molecular Devices, USA). The excitation of Fluo-4 occurs at 490 nm, and the light emission at 524 nm. Records were obtained without interruption for 240 s. The Fluo-4-associated fluorescence intensity was increased by adding histamine (10^−6^ M), and later, verapamil (10^-6^ M) (positive control), HBSS (negative control) or XF-EO were added, in different experiments, to assess the action in modify the [Ca^2+^]_c_. Experiments were performed in triplicate.

### Statistical analysis

The values were expressed as mean and standard error of mean (S.E.M.). Differences between values were statistically compared using Student’s t-test and one-way ANOVA, followed by Bonferroni’s test when applicable, and were considered to differ significantly when *p* < 0.05. IC_50_ and EC_50_ values were calculated with nonlinear regression [29]. All values were analyzed using GraphPad Prism software version 5.01 (GraphPad Software Inc., San Diego CA, USA).

## Results

### Identification of essential oil components

Forty-three compounds of the XF-OE were identified by GC-MS, most of them being represented by monoterpenes (18 %) and sesquiterpenes (67.5 %). The major components were caryophyllene (23.91 %), γ-cadinene (12.48 %), β-ocimene (8.19 %), cadin-4-en-10-ol (5.78 %). δ-cadinene (5.7 %), viridiflorol (4.83 %) and γ-elemene (4.55 %) (Table 1).

**Table 1:** Chemical composition of the essential oil obtained from the leaves of *Xylopia frutescens*

Substances	(Relative %)
Caryophyllene	23,91
γ-cadinene	12,48
β-ocimene	8,19
Cadin-4-en-10-ol	5,78
Viridiflorol	4,83
γ-elemene	4,55
β-elemene	4,31
α-selinene	4,29
Sphatulenol	3,97
Delta-cadinene	3,02
α-humulene	2,48
α –pinene	2,30
γ-muurolene	2,23
β-selinene	2,11
α- cubebene	1,31
Germacrene A	1,25
Aromadendrene	0,99
Camphene	0,94
Mircene	0,92
Bornyl acetate	0,34
Total	90,20

### Effect of XF-EO on carbachol- and histamine-induced phasic contractions

**Fig. 1:**
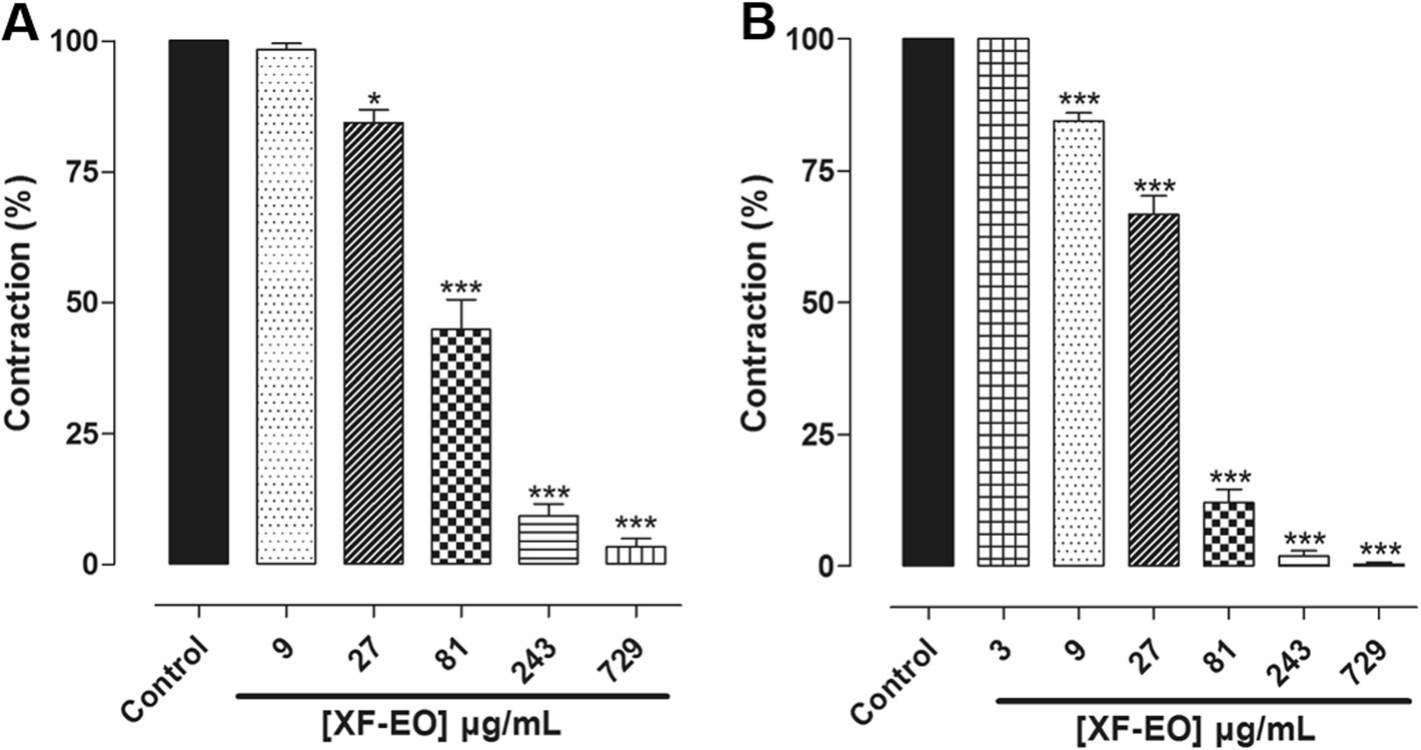
Effect of XF-EO on phasic contractions induced by CCh (**a**) or histamine (**b**) on guinea pig ileum (*n* = 5). Columns and vertical bars represent mean and S.E.M, respectively. One-way ANOVA followed by Bonferroni’s test, *p < 0.05, ***p < 0.001 (control *vs*. XF-EO)

XF-EO (3-729 μg/mL, *n* = 5) antagonized the phasic contractions induced by CCh (IC_50_ = 74.0 ± 8.1 μg/mL) or histamine (IC_50_ = 38.1 ± 2.6 μg/mL) (Fig. 1) on guinea pig ileum in a concentration-dependent manner. The E_max_ values were 97.2 ± 1.3 and 94.6 ± 3.0 %, respectively.

### Effect of XF-EO on histamine-induced cumulative contractions

**Fig. 2:**
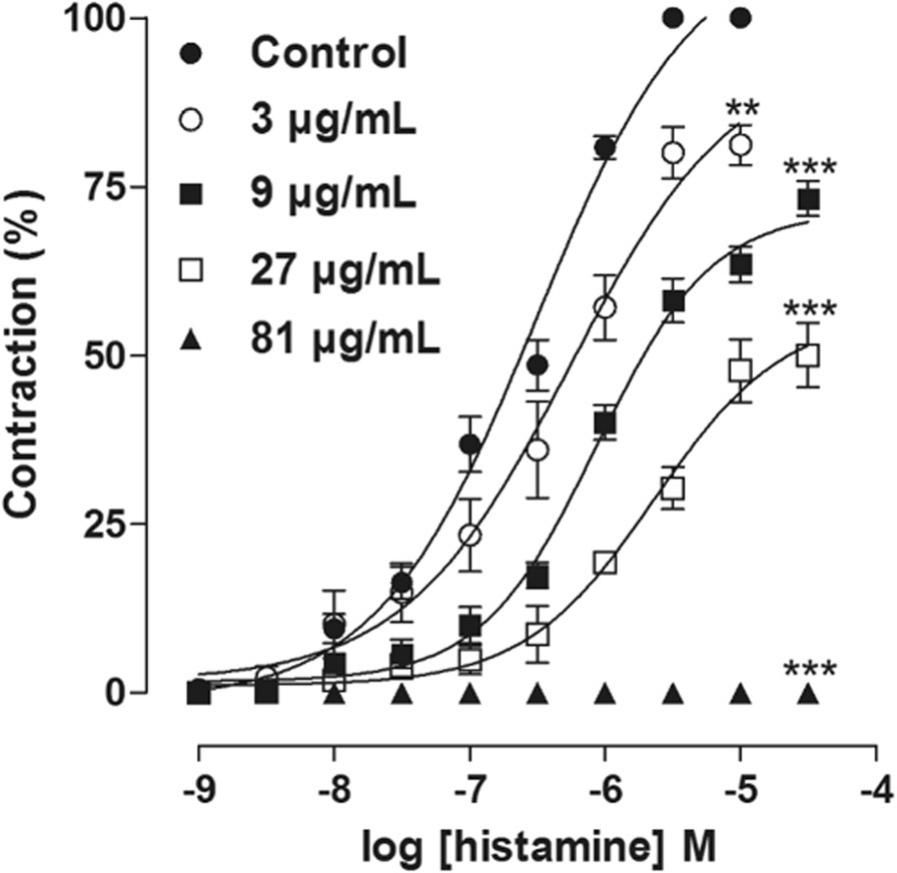
Cumulative concentration-response curves to histamine in both absence (●) and presence of XF-EO: 3 (○), 9 (■), 27 (□) and 81 μg/mL (▲) on guinea pig ileum (*n* = 5). Symbols and vertical bars represent mean and S.E.M, respectively. One-way ANOVA followed by Bonferroni’s test, **p < 0.01, ***p < 0.001 (Control *vs*. XF-EO)

XF-EO (9-81 μg/mL, *n* = 5) concentration-dependently inhibited histamine-induced contractions (Fig. 2). Histamine cumulative concentration-response was non-parallelly shifted to the right and its E_max_ reduced from 100 % (control) to 81.3 ± 2.9, 67.9 ± 3.5, 50.0 ± 4.7 and 0 %. The EC_50_ was attenuated from 2.3 ± 0.3 × 10^−7^ M (control) to 3.8 ± 0.9, 7.3 ± 0.1 × 10^−7^ M and 1.5 ± 0.1 × 10^−6^ M (Fig. 2).

### Effect of XF-EO on KCl-induced tonic contraction or histamine-induced tonic contractions in both absence and presence of non-selective potassium channel blocker

**Fig. 3:**
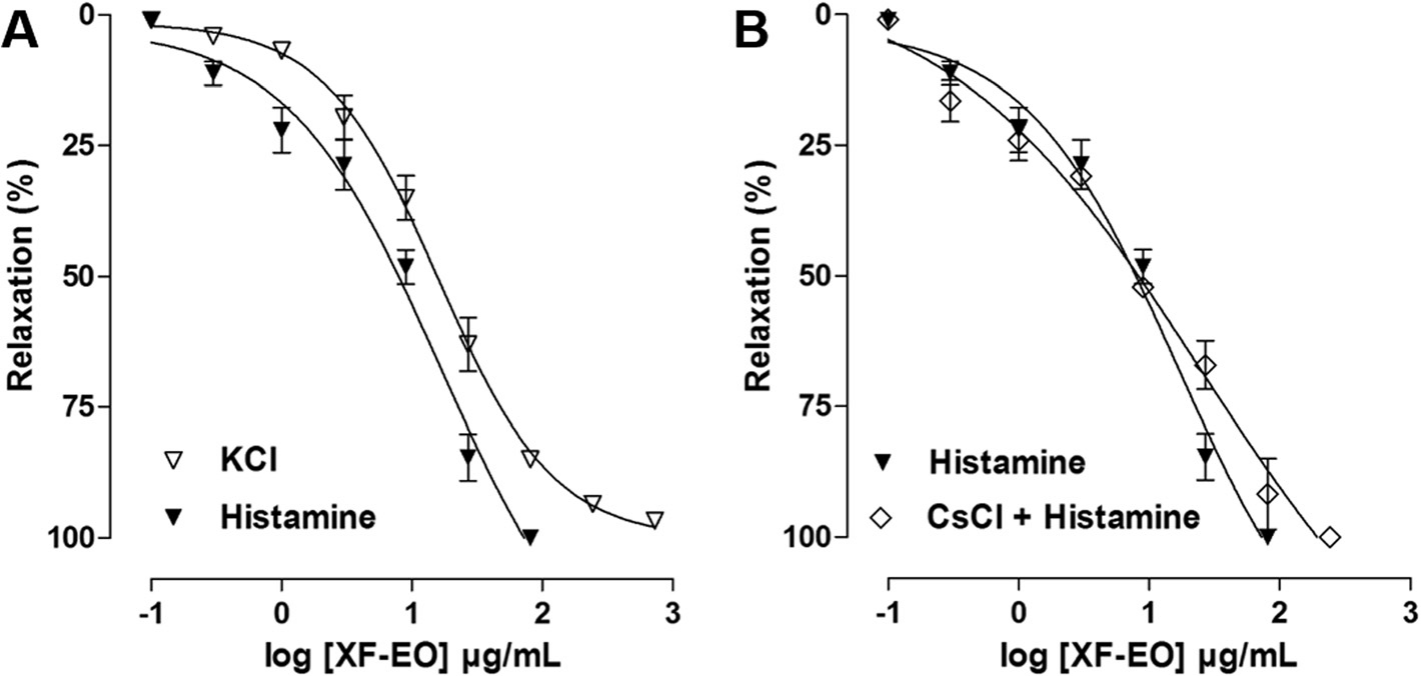
Effect of XF-EO on tonic contractions induced by KCl (40 mM) (∇) or histamine (10^−6^ M) in both absence (▼) (**a**) and presence (◊) of CsCl (5 mM) (**b**) on guinea pig ileum (*n* = 3–5). Symbols and vertical bars represent mean and S.E.M., respectively

XF-EO (0.3–729 or 0.3–81 μg/mL, *n* = 5) showed a concentration-dependent spasmolytic effect on ileum pre-contracted with KCl (EC_50_ = 13.9 ± 1.6 μg/mL) or histamine (EC_50_ = 7.1 ± 0.6 μg/mL) (Fig. 3a). XF-EO relaxant effect was partially reversed 2 h after its removal from organ baths in 62.4 ± 4.2 and 77.8 ± 2.5 %, respectively (data not shown).

XF-EO spasmolytic effect on histamine-induced tonic contraction did not modify statistically in the presence of CsCl (EC_50_ = 7.7 ± 1.2 μg/mL, *n* = 3) (Fig. 3b).

### Effect of XF-EO on Ca^2+^ influx

**Fig. 4:**
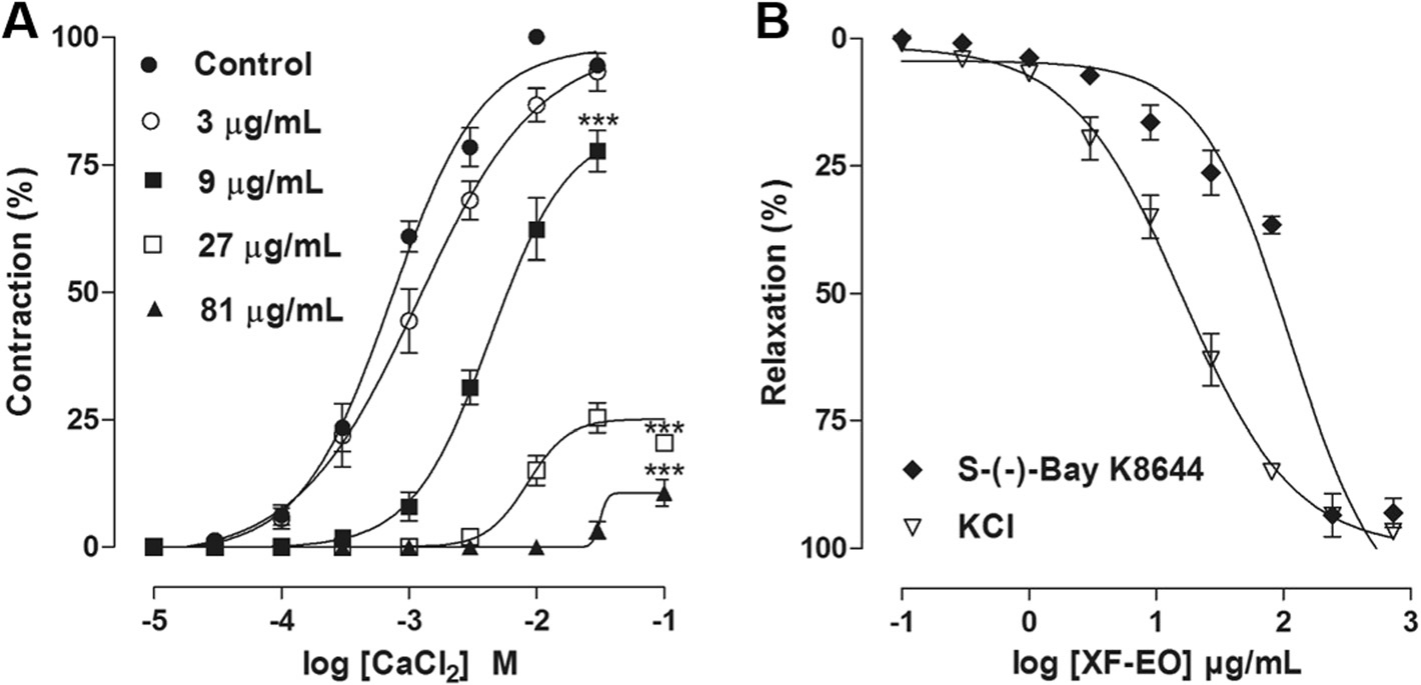
Effect of XF-EO on cumulative curves to CaCl_2_ in absence (●) or presence of 3 (○), 9 (■), 27 (□) and 81 μg/mL (▲) (**a**) or KCl (40 mM) (∇) or S-(-)-Bay K8644 (3 x 10^−7^ M) (♦) tonic contraction (**b**) on guinea pig ileum (*n* = 5). Symbols and vertical bars represent mean and S.E.M, respectively. One-way ANOVA followed by Bonferroni’s test, ***p < 0.001 (Control *vs*. XF-EO)

**Fig. 5:**
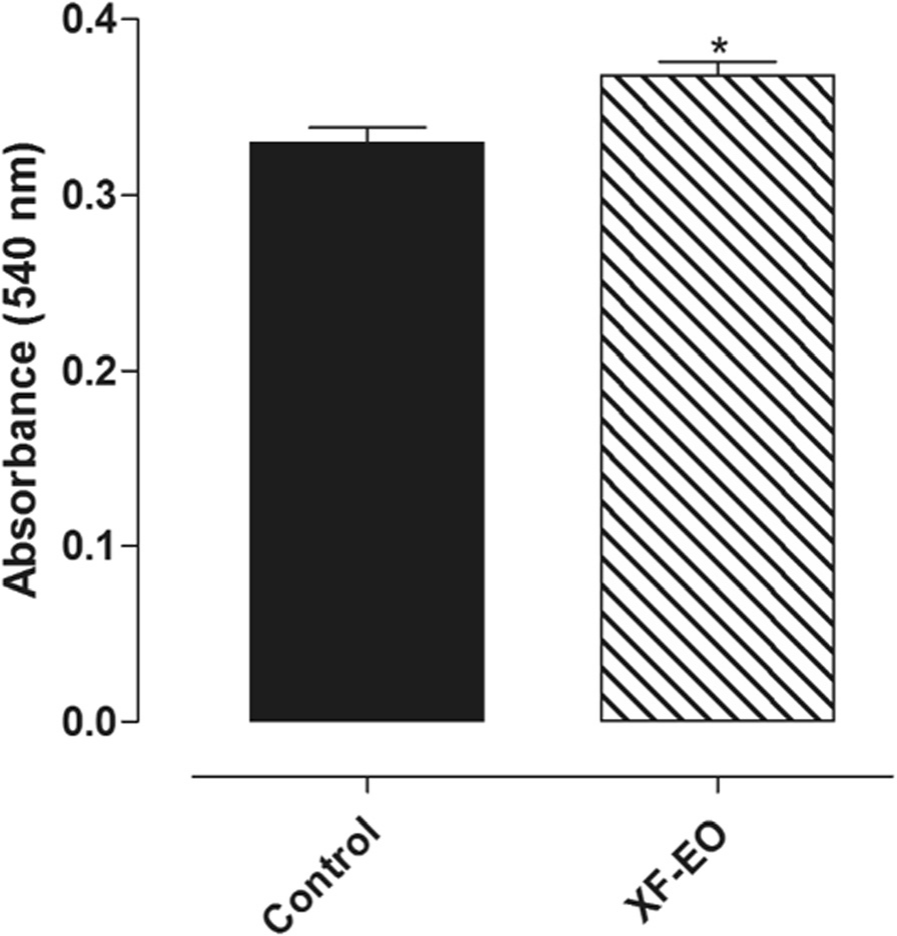
Effect of XF-EO (81 μg/mL) on cell viability of myocytes from the longitudinal layer of guinea pig ileum (*n* = 3). Columns and vertical bars represent mean and S.E.M, respectively. Student’s t-test, *p < 0.05 (Control *vs*. XF-EO)

XF-EO (9-81 μg/mL, *n* = 5) concentration-dependently inhibited Ca^2+^-induced cumulative contractions in a depolarizing medium nominally without Ca^2+^ (Fig. 4a). CaCl_2_ cumulative concentration-response curve was non-parallelly shifted to the right and E_max_ reduced from 100 % (control) to 93.2 ± 3.6, 77.7 ± 4.0, 26.3 ± 2.2 and 10.7 ± 2.6 %. The EC_50_ was attenuated from 0.7 ± 0.1 × 10^−3^ M (control) to 1.0 ± 0.1, 4.0 ± 0.2 and 9.5 ± 1.3 × 10^−3^ M (Fig. 5).

XF-EO (0.1–729 μg/mL, *n* = 5) showed a concentration-dependent spasmolytic effect on ileum pre-contracted with S-(-)-Bay K8644, a selective Ca_V_1 agonist (Fig. 4b). XF-EO was 5 fold more potent in relaxing the ileum pre-contracted with KCl (EC_50_ = 13.9 ± 1.6 μg/mL) than with S-(-)-Bay K8644 (EC_50_ = 74.5 ± 8.2 μg/mL).

### Effect of XF-EO on myocytes from ileum longitudinal layer

#### Interference of XF-EO in the viability of ileum myocytes

XF-EO (81 μg/mL, *n* = 3) did not induced cellular death on ileum myocytes during 2 h of exposure (Fig. 5).

### Effect of XF-EO on cytosolic calcium concentration of ileum myocytes

**Fig. 6:**
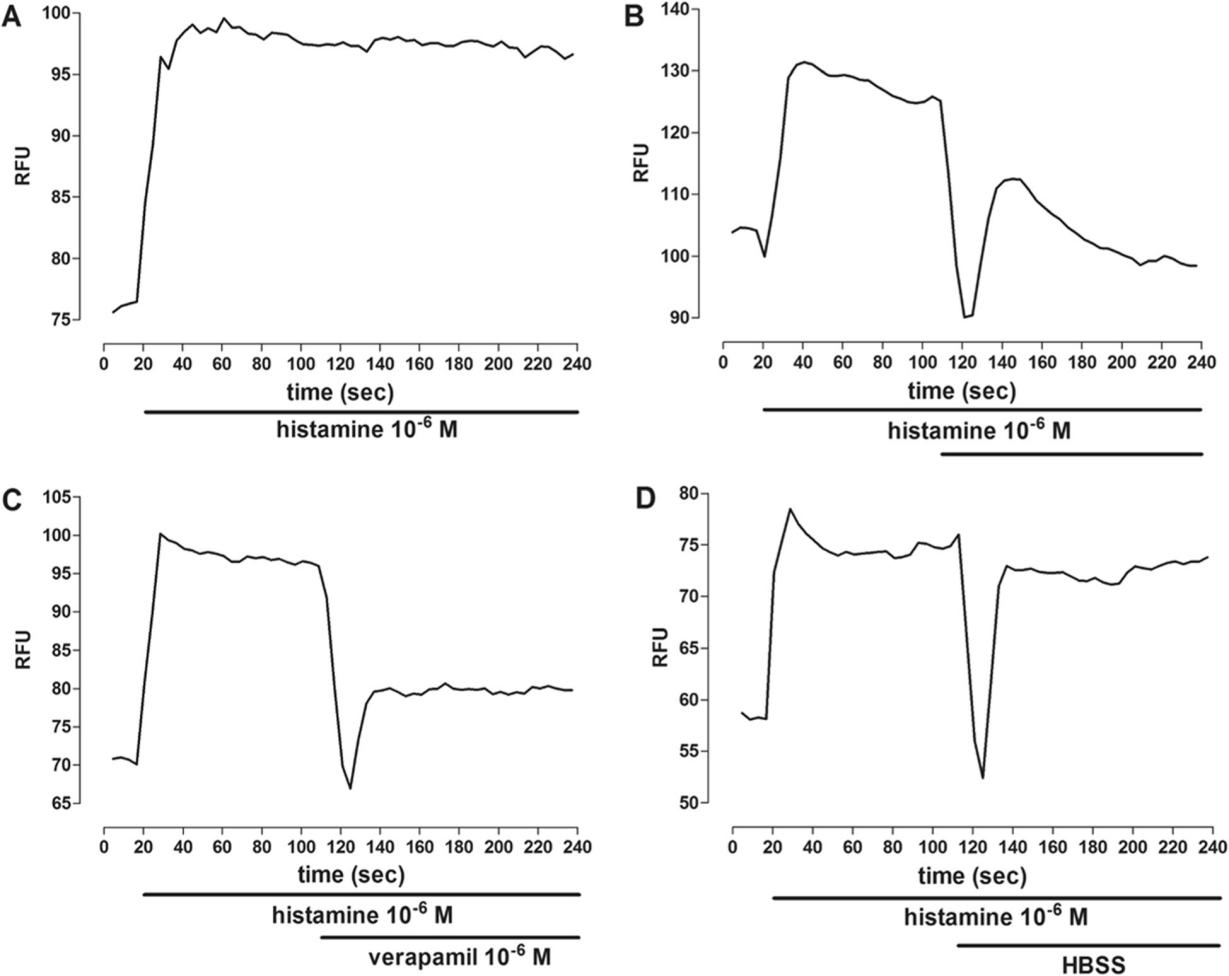
Representative records of control (**a**), XF-EO (81 μg/mL) (**b**), verapamil (10^-6^ M) (**c**) and HBSS (**d**) effects under the sign of calcium in myocytes from the longitudinal layer of guinea pig ileum stimulates with histamine (10^−6^ M) and loaded with Fluo-4

**Fig. 7:**
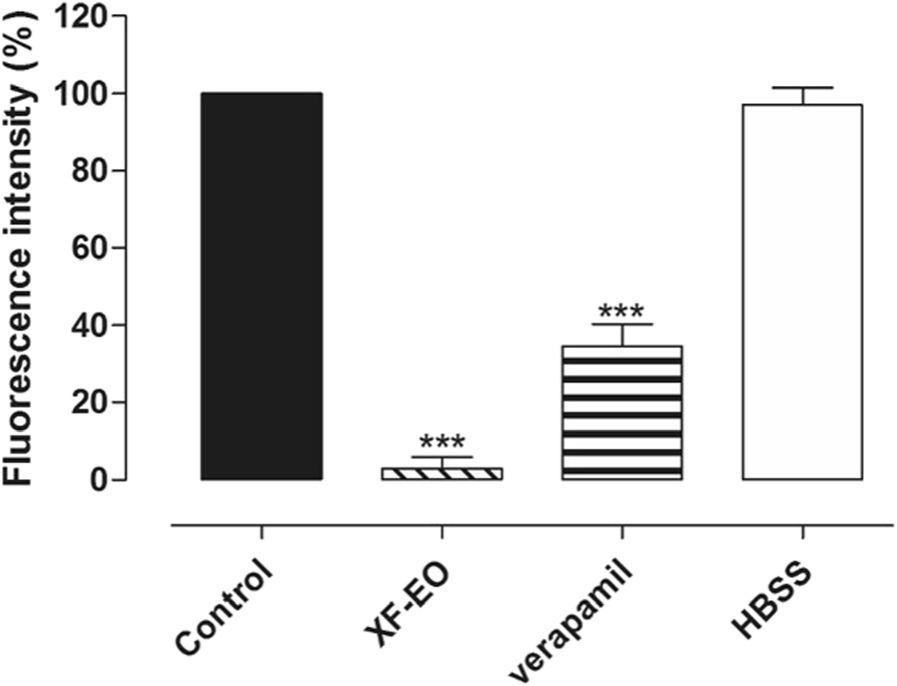
Effect of XF-EO (81 μg/mL) on the Fluo-4-associated fluorescence induced by histamine (10^-6^ M) on guinea pig ileum myocytes (*n* = 3). Columns and vertical bars represent mean and S.E.M., respectively. Student’s t-test, ***p < 0.001 (control *vs*. XF-EO or verapamil)

Histamine (10^−6^ M) induced an increase on Fluo-4-associated fluorescence intensity, remaining stable throughout the stimulation period (220 s) (Figs. 6a and 7). After the stimulation with histamine, at 110 s, XF-EO (81 μg/mL) addition induced a reduction on Fluo-4-associated fluorescence intensity of cells, sustaining this reduction (97.0 ± 3.0 %, *n* = 3) throughout the period of exposure to the essential oil (Figs. 6b and 7). In the same way, verapamil (10^−6^ M), a Ca_V_1 blocker, reduced the [Ca^2+^]_c_ in the initial 10 s, staying reduced (65.5 ± 4.1 %, *n* = 3) throughout the observation period, showing a significant Fluo-4-associated fluorescence intensity decrease (Figs. 6c and 7). The Fluo-4-associated fluorescence decrease showed during the initial 20 s of exposure of the cells to the drugs is caused for the presence of HBSS, as can be demonstrated by a biphasical elevation of the Fluo-4-associated fluorescence intensity after histamine addition, falling slightly and remaining stable throughout the observation period (Figs. 6d and 7).

## Discussion

This study demonstrated for the first time that the essential oil from leaves of *Xylopia frutescens* Aubl. (XF-EO) shows spasmolytic action on guinea pig ileum. Moreover, the most significant finding concerns the characterization of relaxant action mechanism, which includes histaminergic receptor antagonism and possible blockade of Ca_v_1 which reduces cytosolic Ca^2+^ concentration ([Ca^2+^]_c_) leading to smooth muscle relaxation.

Pharmacologic diarrhea therapy include antimotility, spasmolytic, antisecretory, adsorbents, probiotics, anti-infective and miscellaneous agents like α_2_-adrenergic receptor antagonist, Ca^2+^ channel blockers and calmodulin inhibitors [30, 31]. Spasmolytic agents can act by blocking specific receptors present in smooth muscles, such as muscarinic cholinergic or histamine receptors, in a non-specific way, reducing the contractile response from several patterns of stimuli [32].

In the investigation of this study, we detected the presence of spathulenol (1.37 %), considered a marker of essential oils obtained from *Xylopia* genus [33] on the XF-EO. In addition, this essential oil presented a higher percentage of mono- and sesquiterpenes (Table 1). In the pharmacological approach, we found that XF-EO inhibited both CCh- and histamine-induced contractions in a concentration-dependent manner, being more potent in antagonized the histamine-induced contractions (Fig. 1). These data indicate that the essential oil might be acting as a direct antagonist of histamine receptors to promote its spasmolytic effect.

Thus, we investigated the role of histamine receptors using cumulative concentration-response curves to this agonist. XF-EO showed a noncompetitive pharmacologic profile, shifting the histamine cumulative curve nonparallelly to the right with E_max_ reduction (Fig. 2), so the competitive antagonism on histaminergic receptors was refuted. Inhibition of histamine contractile response in XF-EO presence have no shown a limiting value, suggesting a noncompetitive pseudo-irreversible antagonism of H1 receptors. In this particular kind of antagonism, the drug dissociation rate occurs slowly, presenting a prolonged action [34].

Smooth muscle presents a biphasic contraction attributed to a dual source of Ca^2+^ [35, 36]. Particularly, on ileum contraction, the first phase exhibits a fast and transient contraction (phasic component) followed by a long-lasting second phase characterized by the maintained contraction (tonic component) [37, 38]. The removal of extracellular Ca^2+^ prevents contractile responses induced by depolarizing agents (electromechanical coupling), such as KCl, or by agonists of mixed coupling (pharmacomechanical and electromechanical), such as histamine, in few seconds, suggesting that the intracellular Ca^2+^ do not contribute significantly to the tension level [39].

Since the mechanisms involved in tonic contraction maintenance are different from the phasic contraction on guinea-pig ileum [35], we decided to investigate if the essential oil promotes ileum relaxation when pre-contracted with KCl or histamine. XF-EO relaxed this organ pre-contracted with both contractile agents in a concentration-dependent manner, being 2 folds more potent in relaxing the ileum pre-contracted with histamine (Fig. 3a). Partial reverse effect observed can be justified by a noncompetitive pseudo-irreversible antagonism promoted by the essential oil.

Considering that K^+^ channel openers reduce contractile response evoked by low K^+^ concentration and presents potency decreased at high K^+^ concentration [40], another relevant hypothesis is that the essential oil can acts as a K^+^ channel positive modulator. Therefore, in order to verify this hypothesis, XF-EO relaxant effect was assayed in the presence of CsCl, a non-selective K^+^ channel blocker. The presence of CsCl did not alter the essential oil spasmolytic potency (Fig. 3b), suggesting that these channels are not involved in XF-EO action mechanism.

Ileal contractile responses are highly dependent on an increase in free cytoplasmic Ca^2+^. The [Ca^2+^]_c_ increase is due to either influx via Ca_V_ or release from intracellular stores [41]. To test if the essential oil is blocking Ca^2+^ influx through Ca_V_ to promote the spasmolytic action, CaCl_2_ cumulative contractions were induced in a depolarizing medium nominally without Ca^2+^. This protocol is based on the fact that contraction will be obtained almost exclusively by Ca^2+^ from extracellular medium, since depolarization promoted by elevated extracellular K^+^ concentrations leads to Ca_V_ opening [42]. XF-EO inhibited CaCl_2_-induced contractions, shifting the curves to the right and reducing E_max_ (Fig. 4a), reinforcing the hypothesis of Ca^2+^ influx blockade.

Ca_V_ are composed by 4 subunits (2 α, 1 β and 1 γ) where α1 forms the pore that leads to Ca^2+^ influx [43, 44] and are subdivided as Ca_V_1.1, Ca_V_1.2, Ca_V_1.3 and Ca_V_1.4, sensitive to dihydropyridine and high voltage [45]. In smooth muscle, Ca_V_1 are the main responsible to Ca^2+^ influx. Thus, the next step was to confirm and identify the Ca_V_ subtype involved on XF-EO spasmolytic action. Therefore, tonic contractions were obtained with S-(-)-Bay K8644, a specific dihydropyridine derivative agonist for Ca_V_1 that binds directly with α1 subunit to open these channels, but not by depolarization [46]. XF-EO relaxed the ileum pre-contracted with KCl than S-(-)-Bay K8644 (Fig. 4b) suggesting the inhibition of Ca^2+^ influx through Ca_V_1. Meanwhile, the essential oil was more potent in relaxing the ileum pre-contracted with KCl than S-(-)-Bay K8644 (Fig. 4b), suggesting that the Ca^2+^ influx blockade through Ca_V_1 is implicated in the essential oil action mechanism, however other mechanisms seems to be involved in these spasmolytic action, as the inhibited of Ca^2+^ release from intracellular stores [47].

The [Ca^2+^]_c_ decrease is required to promote relaxation on smooth muscle [48, 49]. Nowadays, techniques that use fluorescent indicators allow us to measure the cytosolic Ca^2+^ concentration in several models of smooth muscles [50]. Heretofore, the XF-EO spasmolytic action mechanism theoretically reduces [Ca^2+^]_c_ availability possible due to Ca_V_1 blockade, but no evidence of this has been shown. Thus, we aimed to provide evidence of [Ca^2+^]_c_ reduction by the essential oil in myocytes isolated from ileum longitudinal layer and, so, better characterize its action mechanism. Initially, cell viability was evaluated using the XF-EO maximal concentration used in functional level. In 2 h of XF-EO exposure to the cells was not observed cell death (Fig. 5), this data shows that spasmolytic action is not due to cell death. This way, Fluo-4, a fluorescent indicator of Ca^2+^, was added to the preparations containing ileum myocytes and the resultant Fluo-4-associated fluorescence was quantified in XF-EO presence. The essential oil reduced the great Fluo-4-associated fluorescence emitted in histamine presence, which indicates [Ca^2+^]_c_ reduction of 97 % approximately (Figs. 6 and 7). This reduction is consistent with what was observed in functional level, where maximum relaxation (100 %) was obtained in the 81 μg/mL concentration (Fig. 3a). These results in cellular experiments support our findings in functional level and evidence that XF-EO reduce [Ca^2+^]_c_ to play its spasmolytic role.

## Conclusion

In conclusion, the spasmolytic action of the essential oil from *Xylopia frutescens* Aubl. on guinea pig ileum involves the histaminergic receptor antagonism and possible Ca_V_1 blockade. In addition, it is now established that, in cellular experiments, XF-EO decrease [Ca^2+^]_c_ to promote the spasmolytic action observed in tissue experiments. Further complementary studies on this essential oil *in vivo* protocol are needed to pursue the search for a potential antidiarrheal agent.

## Abbreviations

XF-EO: Essential oil from *Xylopia frutescens* Aubl.
